# Apoptotic vesicles derived from human red blood cells promote bone regeneration via carbonic anhydrase 1

**DOI:** 10.1111/cpr.13547

**Published:** 2023-09-11

**Authors:** Yuzi Shao, Yuhe Jiang, Kunkun Yang, Yuan Zhu, Yunsong Liu, Ping Zhang, Longwei Lv, Xiao Zhang, Yongsheng Zhou

**Affiliations:** ^1^ Department of Prosthodontics Peking University School and Hospital of Stomatology, National Center of Stomatology, National Clinical Research Center for Oral Disease, National Engineering Research Center of Oral Biomaterials and Digital Medical Devices, Beijing Key Laboratory of Digital Stomatology, Research Center of Engineering and Technology for Computerized Dentistry Ministry of Health, NMPA Key Laboratory for Dental Materials Beijing China

## Abstract

Apoptotic vesicles (apoVs) are nanoscale vesicles derived from billions of apoptotic cells involved in the maintenance of the human body's homeostasis. Previous researches have shown that some apoVs, such as those derived from mesenchymal stem cells, contribute to bone formation. However, those apoVs cannot be extracted from patients in large quantities, and cell expansion is needed before apoV isolation, which limits their clinical translation. Mature RBCs, which have no nuclei or genetic material, are easy to obtain, showing high biological safety as a source of extracellular vesicles (EVs). Previous studies have demonstrated that RBC‐derived EVs have multiple biological functions, but it is unknown whether RBCs produce apoVs and what effect these apoVs have on bone regeneration. In this study, we isolated and characterized RBC‐derived apoVs (RBC‐apoVs) from human venous blood and investigated their role in the osteogenesis of human bone mesenchymal stem cells (hBMSCs). We showed that RBCs could produce RBC‐apoVs that expressed both general apoVs markers and RBC markers. RBC‐apoVs significantly promoted osteogenesis of hBMSCs and enhanced bone regeneration in rat calvarial defects. Mechanistically, RBC‐apoVs regulated osteogenesis by transferring carbonic anhydrase 1 (CA1) into hBMSCs and activating the P38 MAPK pathway. Our results indicated that RBC‐apoVs could deliver functional molecules from RBCs to hBMSCs and promote bone regeneration, pointing to possible therapeutic use in bone tissue engineering.

## Graphical abstract

Apoptotic vesicles derived from human red blood cells promoted the osteogenesis of human mesenchymal stem cells and enhanced bone regeneration via carbonic anhydrase I. The figure was created with BioRender.com.

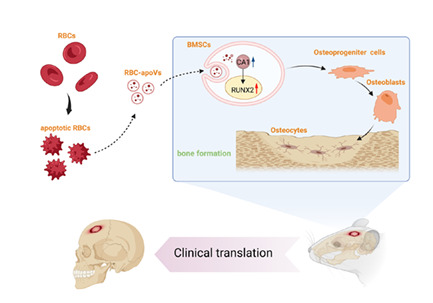

## 1 INTRODUCTION

Bone defects can severely impair patients' physiological function and aesthetic appearance. Bone tissue engineering is an essential strategy for treating oral and maxillofacial bone defects.^1^ In recent years, extracellular vesicles (EVs) represented by exosomes have increasingly been applied in bone tissue engineering as components of cell‐free therapy.^2^, ^3^, ^4^ Exosomes derived from human adipose mesenchymal stem cells (hASCs) have been reported to promote bone regeneration and ameliorate osteoporosis.^5^, ^6^, ^7^ Bone marrow stem cells (BMSCs), osteoblasts and osteocytes can also secrete exosomes capable of promoting osteogenic differentiation.^8^, ^9^, ^10^ Most studies concentrated on the features and application of EVs derived from vital cells, such as exosomes and microvesicles (MVs).^11^ However, the characteristics and functions of vesicles derived from apoptotic cells are still largely unknown.

Apoptosis is a continual activity of cells in the human body involving various pathological processes and physical homeostasis maintenance. A substantial number of apoptotic vesicles (apoVs) are produced by apoptotic cells every day.^12^, ^13^ Mounting evidence suggests that apoVs differ from EVs produced by healthy cells in terms of cargoes, features, functions and producing mechanisms. Therefore, apoVs are emerging as promising components in cell‐free therapy.^14^, ^15^ Our previous study has shown that macrophage‐derived apoptotic vesicles could regulate the osteogenesis and adipogenesis of mesenchymal stem cells (MSCs). They also contribute to liver macrophage homeostasis restoration.^16^, ^17^ ApoVs derived from MSCs (MSC‐apoVs) have been shown to exert therapeutic effects on multiple diseases, such as osteopenia and haemophilia.^15^, ^18^ Nevertheless, there are limitations of MSC‐apoVs in terms of clinical application. For example, parental cells for MSC‐apoVs extraction are usually purchased rather than obtained directly from patients, and MSCs must be expanded in vitro before apoptotic induction.^19^ Besides, the apoVs derived from nucleated cells contain DNA that will inevitably be delivered to the recipient cells.^20^ Conventional drugs used for apoptosis induction, such as staurosporine (STS), are usually cytotoxic.^18^ Therefore, MSC‐apoVs still present a biosafety risk. A safer and more efficient source of apoVs is needed for clinical transformation.

Red blood cells (RBCs) are the most numerous cells and one of the most frequently renewing cells in the human body since large numbers of RBCs undergo apoptosis every day. A series of studies have indicated that RBCs can secrete vesicles during erythropoiesis, physiological cell aging, pathological processes and stress‐induced conditions.^21^, ^22^ RBCs can be obtained from patients conveniently, and cell expansion is unnecessary before vesicle isolation, making RBCs an appealing option for apoV production and clinical transformation. Since mature RBCs do not contain nuclei and organelles, their apoptosis differs from that of nucleated cells. Many stimuli, such as oxidative stress and osmotic shock, can cause apoptosis of RBCs in vivo and in vitro.^23^, ^24^, ^25^ RBC‐derived exosomes and MVs contain active components such as proteins, lipids and miRNAs.^26^, ^27^ Several studies have shown that RBC‐derived EVs have procoagulant and immunomodulatory effects. In addition, they have the potential to serve as drug delivery vectors and as clinical diagnostic markers for a variety of disorders related to RBCs.^28^, ^29^, ^30^, ^31^ However, the properties and applications of RBC‐derived apoVs are currently unclear.

In this study, we characterized apoVs derived from RBCs (RBC‐apoVs) and investigated their promotional effect on the osteogenesis of human BMSCs (hBMSCs) and bone regeneration. Proteomic analysis results demonstrated that carbonic anhydrase 1 (CA1), a highly enriched protein in RBCs,^32^ could be transferred to hBMSCs. Additionally, we further explored the influence of CA1 on the osteogenic differentiation of hBMSCs in vitro.

## 2 MATERIALS AND METHODS

### 2.1 Collection and apoptotic induction of RBCs

This study was approved by the Medical Ethics Committee of the Peking University School and Hospital of Stomatology (PKUSSIRB‐202170183). Fresh human venous blood was collected from healthy donors at the Peking University Hospital of Stomatology. The donors were of both sexes, aged above 18 years. The blood was obtained by venipuncture using blood collection tubes containing 3.8% sodium citrate (Greiner, German). The volume of blood we collected was 2 mL per individual. Then, the whole blood was centrifuged at 300 *g* for 10 min at room temperature (RT) to remove the buffy coat and plasma. Fresh RBCs were rinsed in phosphate buffered solution (PBS) for three times. Subsequently, RBCs were mixed with RBC lysis buffer (Solarbio, China) at a volume ratio of 1:6 and placed on ice for 30 min to induce RBC apoptosis.

### 2.2 Isolation and purification of RBC‐apoVs

After lysis, the RBC lysate mixture was centrifuged at 800 *g* for 10 min at 4°C. The supernatant was then moved to a clean centrifuge tube, followed by centrifugation at 16,000 *g* for 30 min at 4°C. After centrifugation, we aspirated and discarded the supernatant and washed the sediment with 0.22 μm‐filtered PBS to obtain RBC‐apoVs. A Pierce BCA Protein Assay Kit (Thermo Scientific) was used to measure the concentration of RBC‐apoVs.

### 2.3 Identification of RBCs, apo‐RBCs and RBC‐apoVs

We performed the field emission scanning electron microscopy (SEM) observation to get morphology images of fresh RBCs and apoptotic RBCs (apo‐RBCs) treated with RBC lysis buffer for 10 min and 30 min. Cells were fixed using 2.5% glutaraldehyde fixative. They were resuspended in Milli Q water, placed onto the silica wafers, and dried with silica gel desiccant overnight at RT. After sputter coating with gold, the samples were viewed with a Hitachi SU‐8010 SEM (Hitachi, Japan).

Using flow cytometry, surface markers of RBCs, apo‐RBCs, and RBC‐apoVs were identified. The cell surface staining and apoV surface staining was conducted as previously described.^15^, ^19^ Phosphatidylserine (PS) was detected with an Annexin V‐FITC Apoptosis Detection Kit (Beyotime, China) to evaluate apoptosis. RBC‐apoVs, apo‐RBCs treated with lysis buffer for 30 min and fresh RBCs were collected, suspended in Annexin V binding buffer and stained for 20 min at 4°C with FITC‐Annexin V. CD235a, also referred to as Glycophorin A, is a major sialoglycoprotein expressed on the membrane of human RBCs, which is frequently used for the identification of RBCs.^33^ For identification of surface markers on human RBCs, all cells and apoVs were treated with PE‐conjugated anti‐human CD235a (BioLegend) at 4°C for 30 min. After incubation, cells and apoVs were rinsed twice before being analysed with a NovoCyte flow cytometer (ACEA Biosciences). FlowJo 10.4 (FlowJo) software was utilized for data analysis. A Western blot was performed to detect common EVs markers using anti‐CD81, anti‐TSG101 and anti‐GAPDH antibodies. The detailed procedures of Western blotting are stated in Section 2.15.

Nanoparticle tracking analysis (NTA) was conducted with a ZetaView PMX120 (Particle Metrix, Germany) to evaluate the size distribution and concentration of RBC‐apoVs. RBC‐apoVs were resuspended and diluted in PBS. Then, the particle size distribution and potential of RBC‐apoVs were measured. The ZetaView software (version 8.05.14 SP7) was used for data analysis. We also quantified the protein concentration of RBC‐apoVs using a Pierce BCA Kit.

The morphology images of RBC‐apoVs were examined using transmission electron microscopy (TEM). RBC‐apoVs were fixed and air‐dried before being negatively stained twice with 1% uranyl acetate. We used a JEM‐1400PLUS TEM (JEOL, Japan) to obtain images.

### 2.4 Culture and osteogenic induction of hBMSCs

Primary hBMSCs were purchased from ScienCell Research Laboratories (USA). The cells were cultured in proliferation medium (PM) containing α‐minimum essential medium (α‐MEM, Gibco), 10% (v/v) foetal bovine serum (FBS, Gibco) and 1% (v/v) penicillin streptomycin (Gibco). The osteogenic induction medium (OM) contained 10% FBS, 1% penicillin streptomycin, 10 nM dexamethasone (Sigma‐Aldrich), 200 μM ascorbic acid (Sigma‐Aldrich) and 10 mM β‐glycerophosphate (Sigma‐Aldrich) in α‐MEM.

### 2.5 RBC‐apoV uptake by hBMSCs in vitro

RBC‐apoVs were labelled with PKH‐26 (Sigma‐Aldrich) following the manufacturer's instructions. Next, the labelled RBC‐apoVs were incubated with hBMSCs for 12, 24 and 48 h. Then, hBMSCs were rinsed and fixed with 4% paraformaldehyde at RT. After 10 min, hBMSCs were washed with PBS, treated with 0.1% Triton X‐100 (Sigma‐Aldrich) at RT for 7 min and then washed again. F‐actin was stained with FITC‐Cyclopeptide (Sigma‐Aldrich). 6‐diamidine‐2‐phenylindole (DAPI) was used to stain nuclei. A Zeiss LSM880 confocal microscope (Zeiss, Germany) was used to acquire the images.

### 2.6 Cell proliferation assay

We used a Cell Counting Kit‐8 (CCK‐8, Dojindo, Japan) to detect cell proliferation. Briefly, hBMSCs were seeded in 96‐well plates at a density of 2000 cells/well and then exposed to PM, PM with 0.10 μg/mL RBC‐apoVs, PM with 0.25 μg/mL RBC‐apoVs, PM with 0.50 μg/mL RBC‐apoVs and PM with 0.75 μg/mL RBC‐apoVs, respectively. Cells were tested daily, and the absorbance was detected at 450 nm (*n* = 3).

### 2.7 Alizarin red S staining and quantification

To determine the optimal concentration of RBC‐apoVs, hBMSCs were divided into six groups: (1) PM, (2) OM, (3) OM with 0.10 μg/mL RBC‐apoVs, (4) OM with 0.25 μg/mL RBC‐apoVs, (5) OM with 0.50 μg/mL RBC‐apoVs and (6) OM with 0.75 μg/mL RBC‐apoVs. After 10 days of osteogenic induction, hBMSCs were rinsed with PBS and fixed with 95% ethanol for 30 min. The cells were stained with 1% alizarin red S (ARS) solution (Sigma‐Aldrich). For the matrix calcification quantification, the stained cells were treated with 100 nM cetylpyridine solution for 30 min. Then, the absorbance at 562 nm was examined. Based on the results, we chose 0.50 μg/mL RBC‐apoVs as the optimal concentration in the following experiments. We further examined the effect of RBC‐apoVs on the osteogenic differentiation of hBMSCs at 5 and 10 days of induction.

### 2.8 Alkaline phosphatase staining and activity

To examine early markers of osteogenic differentiation, alkaline phosphatase (ALP) staining and ALP activity quantification were conducted. The hBMSCs were divided into three groups: (1) PM, (2) OM and (3) OM with RBC‐apoVs. After 5 days of induction, we performed ALP staining using a BCIP/NBT Staining Kit (Beyotime Biotechnology, China). Meanwhile, we quantified the ALP activity with an ALP Activity Assay Kit (Nanjing Jiancheng, China) according to the manufacturer's protocol. The absorbance at 520 nm was detected. ALP activity normalized to the total protein content was calculated.

### 2.9 In vivo implantation of hBMSCs

Animal experiments were approved by the Institutional Animal Care and Use Committee of the Peking University Health Science Center (LA2021456). Six‐week‐old female BALB/C nude mice were purchased from Vital River Corporation (Beijing, China). hBMSCs were grown in PM and PM with RBC‐apoVs for a week for heterotopic bone formation. The cells were collected and mixed with β‐tricalcium phosphate (β‐TCP) (Rebone Biomaterials, China) scaffolds at 37°C for 1 h. Then, the mixtures were subjected to centrifugation at 150 *g* for 5 min. Next, nude mice were divided into three groups according to different materials implanted in their dorsal subcutaneous space: (1) β‐TCP scaffold only (β‐TCP group), (2) hBMSCs/β‐TCP composite scaffold construction (hBMSCs group), and (3) hBMSCs treated with RBC‐apoVs/β‐TCP composite scaffold (hBMSCs + RBC‐apoVs group). Each site was inoculated with a total of 1 × 10^6^ cells. After 8 weeks, we collected and analysed the samples using haematoxylin–eosin (H&E) and Masson staining.

### 2.10 Rat calvarial defect model

According to our previous studies, poly(lactic‐co‐glycolic acid) (PLGA) scaffolds modified with polydopamine (pDA) were constructed as the carrier of RBC‐apoVs.^6^ Briefly, PLGA (lactide/glycolide: 50/50) scaffolds purchased from the Shandong Academy of Pharmaceutical Sciences (Shandong, China) were prepared as cylinders of 5 mm in diameter and 2 mm in height. We soaked the PLGA scaffolds in dopamine (DA, Sigma‐Aldrich) solution (2 mg/mL in 10 mM Tris–HCl, pH 8.5) to form the pDA film. Next, scaffolds were washed in PBS, subjected to sterilization with 75% ethanol, and rewashed. For immobilization of RBC‐apoVs, the PLGA scaffolds and PLGA/pDA scaffolds were subjected to incubation with RBC‐apoVs at 4°C for 12 h. To measure the amount of RBC‐apoVs released, the materials loaded with RBC‐apoVs were placed in stroke‐physiological saline solution for 7 days at 37°C. The supernatants were obtained daily, and the concentration of RBC‐apoVs in saline solution was measured using a Pierce BCA Kit. To observe the surface morphology of the scaffolds, PLGA, PLGA/pDA and PLAG/pDA with RBC‐apoVs were fixed in 4% glutaraldehyde for 12 h at 4°C. All scaffolds were then dried in a critical point dryer (Micro Modul YO‐230, Thermo Scientific). Images were captured using a SU‐8010 SEM (Hitachi, Japan).

After the construction of PLGA/pDA scaffolds and RBC‐apoVs' immobilization, 8‐week‐old male rats (Vital River, China) were divided into three groups: (1) blank; (2) PLGA/pDA scaffolds only (PLGA/pDA); (3) PLGA/pDA with RBC‐apoVs (PLGA/pDA + RBC‐apoVs). Calvarial skull defect experiments were performed, and 5 mm‐diameter critical‐sized defects were made with a trephine bur under low‐speed drilling. The compounds were implanted into the defect areas.

### 2.11 Analysis of bone formation in vivo

After 8 weeks of implantation, the rats were executed. The calvaria specimens, which included the implants, were collected and fixed in 4% paraformaldehyde. The samples were scanned using a high‐resolution Inveon micro‐computed tomography (micro‐CT; Siemens, Germany). Using the Inveon Research Workplace (Siemens, Germany) software, a three‐dimensional reconstruction and parametric analysis were conducted for the evaluation of bone formation within the bone defects. Following that, H&E and Masson staining were performed after decalcification.

### 2.12 Proteomic analysis

For sample preparation, hBMSCs were cultured in OM and OM with RBC‐apoVs, respectively. After 7 days of osteogenic induction, protein lysates from the two groups of hBMSCs were prepared. The nano‐LC–MS/MS analysis was performed using a nano‐UPLC (EASY‐nLC1200, Thermo Fisher Scientific) linked to a Q Exactive HF‐X Orbitrap apparatus (Thermo Fisher Scientific). Data from mass spectrometry (MS) were obtained using the data‐dependent acquisition mode. Proteome Discoverer software (Version 2.4.0.305, Thermo Fisher Scientific) and the built‐in Sequest HT search engine were used to process the raw MS files. Proteins were identified by comparing them to the UniProt FASTA databases, with the false discovery rate for both peptides and proteins set to 0.01. For protein quantification, unique peptides and razor peptides were employed. The total peptide amount was used for normalization. Proteins that were differentially expressed (fold change <0.83 or >1.2; *p* value < 0.05) were included for subcellular localization analysis, clusters of orthologous groups for eukaryotic complete genomes (KOG) analysis and further functional analysis based on the Gene Ontology (GO) databases and the Kyoto Encyclopedia of Genes and Genomes (KEGG) database.

### 2.13 Small interfering RNA (siRNA) transfection

Sangon Biotech (Shanghai, China) provided the *CA1*‐targeting small interfering RNA (siRNA) (si*CA1*) and the non‐targeting control siRNA (NC). The siRNA sequences we used are displayed in Table 1. Lipofectamine 3000 (Invitrogen) was used to transfect the hBMSCs with siRNA. After 48 h, the hBMSCs were collected for RNA and protein analysis to detect transfection efficiency. For osteogenic differentiation, the cells were transfected in OM. They were collected after 10 days of induction. ALP staining and ARS staining were performed for osteogenic assessment.

**TABLE 1:** Sequences of siRNAs.

siRNA	Sense (5′ to 3′)	Antisense (5′ to 3′)
si*CA1*‐1	CGCAGCCUUCUAUCAAAUGUUTT	AACAUUUGAUAGAAGGCUGCGTT
si*CA1*‐2	CGAUAACCGAUCAGUGCUGAATT	UUCAGCACUGAUCGGUUAUCGTT
NC	UUCUCCGAACGUGUCACGUTT	ACGUGACACGUUCGGAGAATT

### 2.14 Real‐time quantitative PCR

We extracted total RNA from hBMSCs using the TRIzol reagent (Invitrogen). To acquire cDNA, reverse transcription was conducted using a PrimeScript RT Reagent Kit (Takara, Japan). Next, the gene expression level was tested using a FastStart Universal SYBR Green Master Mix (Roche Applied Science, Germany) and a 7500 Real‐Time PCR System (Applied Biosystems). The reference gene was glyceraldehyde‐3‐phosphate dehydrogenase (*GAPDH*). Table 2 shows the primer sequences.

**TABLE 2:** Sequences of the primers.

Gene	Forward (5′ to 3′)	Reverse (5′ to 3′)
*GAPDH*	CGGACCAATACGACCAAATCCG	AGCCACATCGCTCAGACACC
*ALP*	GACCTCCTCGGAAGACACTC	TGAAGGGCTTCTTGTCTGTG
*RUNX2*	TCTTAGAACAAATTCTGCCCTTT	TGCTTTGGTCTTGAAATCACA
*COL1A1*	ACAGGGCTCTAATGATGTTGA	AGGCGTGATGGCTTATTTGT
*BSP*	CAGGCCACGATATTATCTTTACA	CTCCTCTTCTTCCTCCTCCTC
*OCN*	CACTCCTCGCCCTATTGGC	CCCTCCTGCTTGGACACAAAG
*OPN*	ATGATGGCCGAGGTGATAGT	ACCATTCAACTCCTCGCTTT
*CA1*	GCTACAGGCTCTTTCAGTT	GACTCCATCCACTGTATGTT

### 2.15 Western blotting

To prepare lysates of cells or RBC‐apoVs, radioimmunoprecipitation assay (RIPA) lysis buffer (Huaxingbio, China) with 1% protease and phosphatase inhibitor cocktail (NCM Biotech, China) was used. Protein extraction was performed by centrifugation of the lysates. We used a Pierce BCA Kit to determine the protein concentration. The extracts of proteins were loaded onto 10% SDS–PAGE and transferred to polyvinylidene fluoride membranes. The membranes were blocked and incubated with primary antibodies at 4°C for 12 h, and then treated with secondary antibody (1:10,000; Huaxingbio, China). Subsequently, we used an enhanced chemiluminescence kit (NCM Biotech, China) to visualize protein bands. The primary antibodies were as follows: anti‐CD81 (1:1000; Abcam, UK), anti‐TSG101 (1:1000; Abcam, UK), anti‐RUNX2 (1:1000; Abcam, UK), anti‐CA1 (1:500; Abcam, UK), anti‐p38 (1:500; ZenBio, China), anti‐Phospho‐p38 (1:500; ZenBio, China) and anti‐GAPDH (1:5000; Huaxingbio, China).

### 2.16 Statistical analysis

The data were presented as mean ± standard deviation (SD). Statistical analyses were performed with GraphPad Prism 8.0 (GraphPad Software). Comparisons between two groups were performed using independent two‐tailed Student's *t* tests. One‐way ANOVA and a Tukey's post hoc test were used for multiple groups' comparisons. *P* value < 0.05 were considered statistically significant.

## 3 RESULTS

### 3.1 Isolation and characterization of RBCs, apo‐RBCs and RBC‐apoVs

**FIGURE 1:**
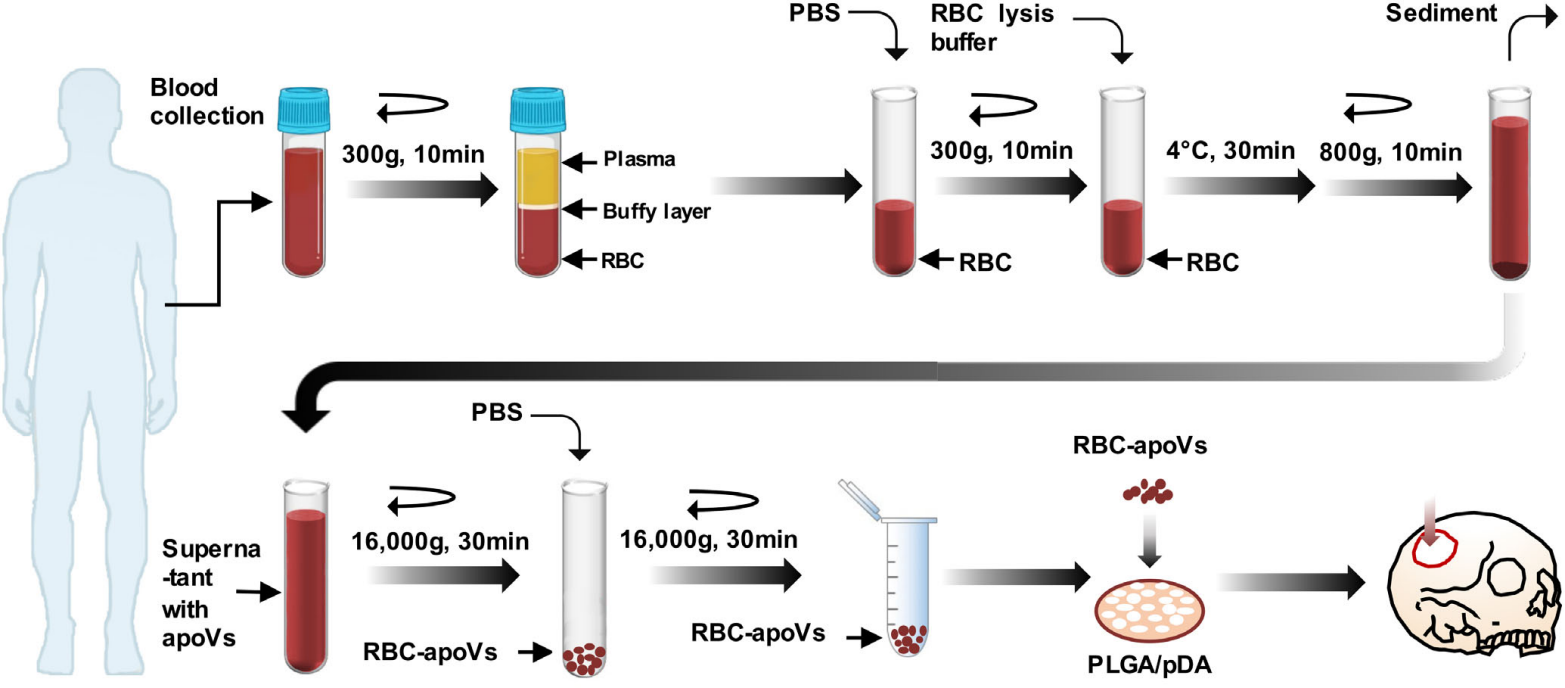
Isolation and application of red blood cell‐derived apoptotic vesicles (RBC‐apoVs). Schematic diagram indicating the procedures of isolating RBC‐derived apoVs from human venous blood and the therapeutic application of RBC‐apoVs. apoVs, apoptotic vesicles; PBS, phosphate buffered solution; PLGA/pDA, poly(lactic‐co‐glycolic acid) scaffolds modified with polydopamine; RBCs, red blood cells.

**FIGURE 2:**
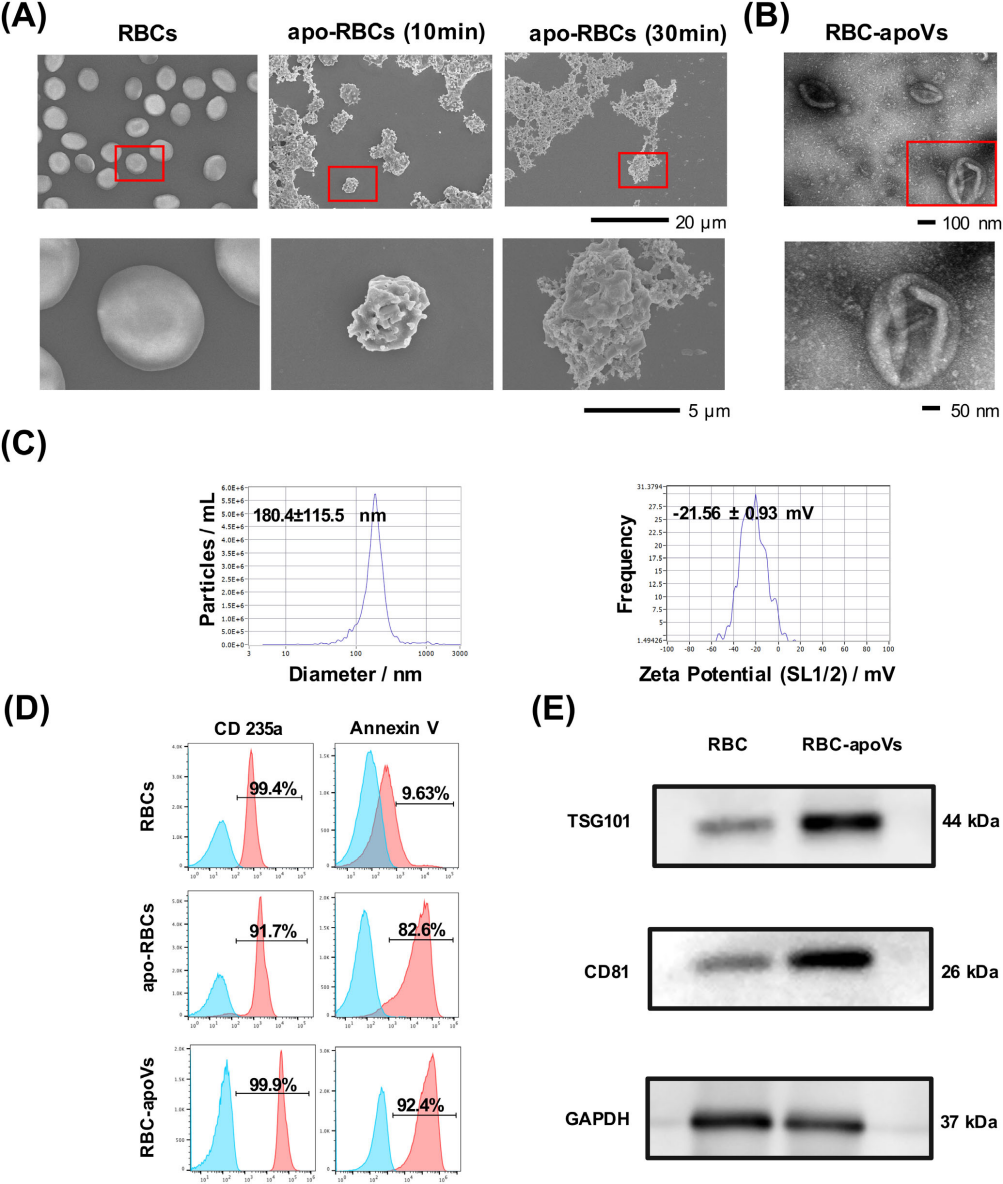
Characteristics of RBCs, apo‐RBCs and RBC‐apoVs. (A) Morphology of RBCs and RBCs treated with lysis buffer for 10 min (apo‐RBCs [10 min]) and 30 min (apo‐RBCs [30 min]) by SEM. The red rectangles indicated the corresponding magnified areas. (B) Morphology of RBC‐apoVs by TEM. (C) Nanoparticle tracking analysis by Zetaview revealed the particle size distribution and membrane potential of RBC‐apoVs: the mean size ± SD was 180.4 ± 115.5 nm; the mean potential ± SD was −21.56 ± 0.93 mV. (D) Flow cytometric analysis revealing surface marker expression of RBC‐apoVs. (E) Western blotting analysis of RBCs and RBC‐apoVs. apo‐RBCs, apoptotic RBCs; RBC‐apoVs, RBC‐derived apoVs; SEM, scanning electron microscopy; TEM, transmission electron microscopy.

We isolated RBCs from human venous blood and induced the apoptosis of RBCs. RBC‐apoVs were isolated from apo‐RBCs using an optimized gradient centrifugation protocol (Figure 1). SEM was employed to observe the morphologic change between RBCs and apo‐RBCs. Freshly obtained RBCs were biconcave disc‐shaped cells with a diameter of 6–8 mm, while apo‐RBCs treated with lysis buffer after 10 min shrank obviously. A large number of vesicle‐like protrusions appeared on the surface of apo‐RBCs. After treatment with lysis buffer for 30 min, apo‐RBCs lost their shapes completely, showing apparent cell aggregation and adhesion (Figure 2A). RBC‐apoVs were cup‐shaped vesicles with a diameter of around 200 nm, as revealed by TEM (Figure 2B). The diameter distribution and membrane potential of RBC‐apoVs were shown by NTA (Figure 2C). According to the NTA results, 1.2E + 11 particles of RBC‐apoVs could be isolated per individual. In addition, based on the BCA results, about 14.7 mg RBC‐apoVs could be obtained per individual. Surface markers of RBCs, apo‐RBCs and RBC‐apoVs were analysed by flow cytometry (Figure 2D). CD235a is a common surface indicator of RBCs.^34^ Our results confirmed that both RBCs and apo‐RBCs had high expression of CD235a. Similar to their parental cells, RBC‐apoVs also highly expressed CD235a. The surface exposure of PS, a symbol of apoptotic cells and apoVs, was examined by Annexin V binding. Flow cytometry analysis indicated that after treatment with lysis buffer for 30 min, apo‐RBCs had smaller sizes and a higher level of PS exposure than normal RBCs. RBC‐apoVs had a 92.4% Annexin V positivity rate. Western blot analysis revealed that RBC‐apoVs expressed elevated levels of the EV biomarkers TSG101 and CD81 compared with RBCs (Figure 2E). The above results showed that apo‐RBCs also present the features like other apoptotic cells, including cell shrinkage, plasma membrane blebbing and PS externalization. Additionally, RBC‐apoVs matched the apoVs' membrane surface features and exhibited the plasma membrane characteristics of RBCs.

### 3.2 Internalization of RBC‐apoVs by hBMSCs

**FIGURE 3:**
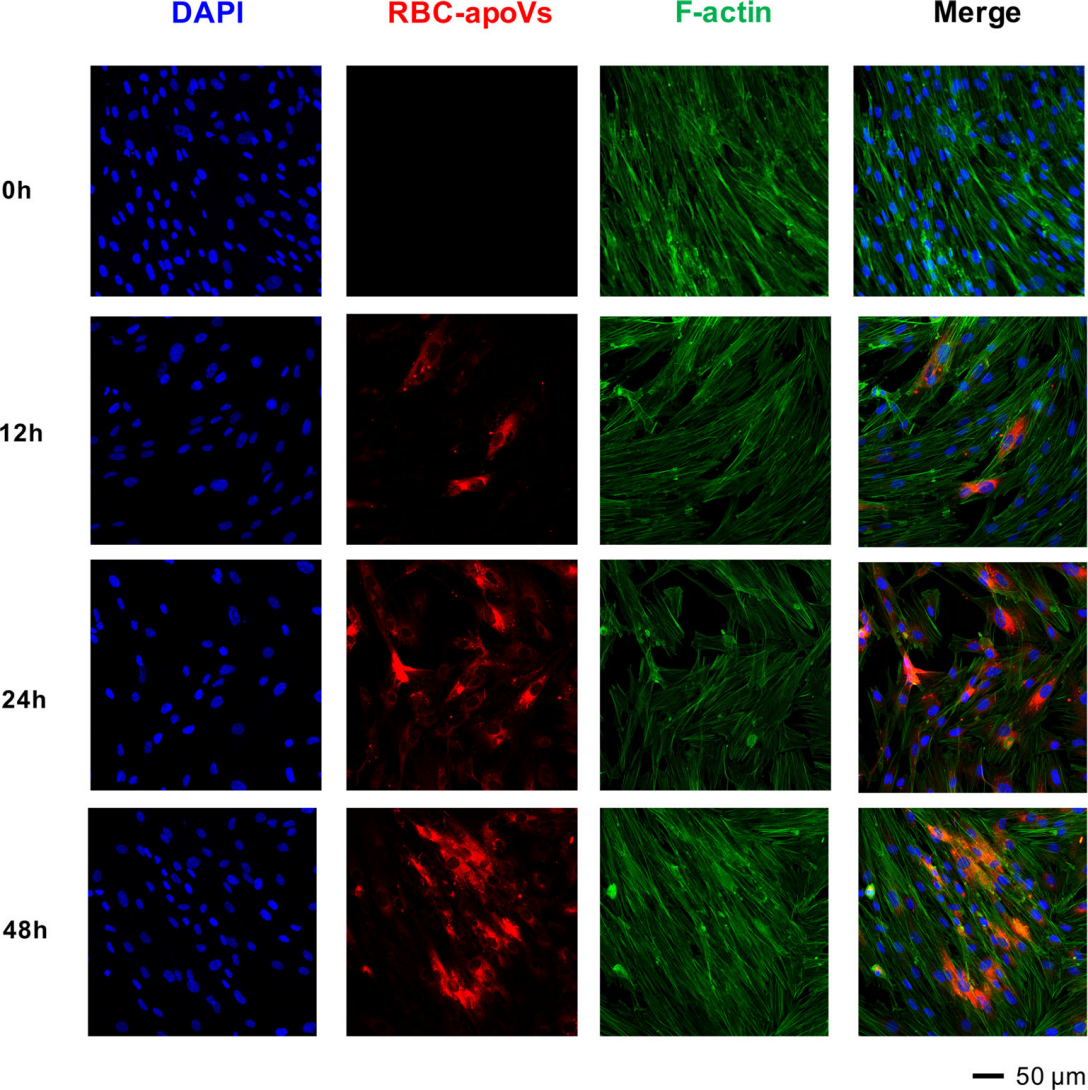
RBC‐apoVs were internalized by hBMSCs. RBC‐apoVs were labelled with PKH‐26 (red). The nuclei of hBMSCs were stained with DAPI (blue). The F‐actin of hBMSCs was stained with phalloidin (green). DAPI, 6‐diamidine‐2‐phenylindole; hBMSCs, human bone marrow mesenchymal stem cells; RBC‐apoVs, RBC‐derived apoVs.

To detect whether hBMSCs could internalize RBC‐apoVs, hBMSCs were incubated with RBC‐apoVs stained by PKH‐26 (red) for 12, 24 and 48 h. The F‐actin of hBMSCs was labelled with phalloidin (green). The nuclei were labelled with DAPI (blue). Confocal laser microscopy images demonstrated that RBC‐apoVs (the red fluorescence) started to accumulate near the nuclei from 0 to 12 h. From 12 to 24 h, the number of red‐stained particles gradually increased, which meant increasing RBC‐apoVs were internalized by hBMSCs. A significant number of RBC‐apoVs were detected in the perinuclear region 48 h after incubation (Figure 3).

### 3.3 RBC‐apoVs promoted osteogenic differentiation of hBMSCs in vitro

**FIGURE 4:**
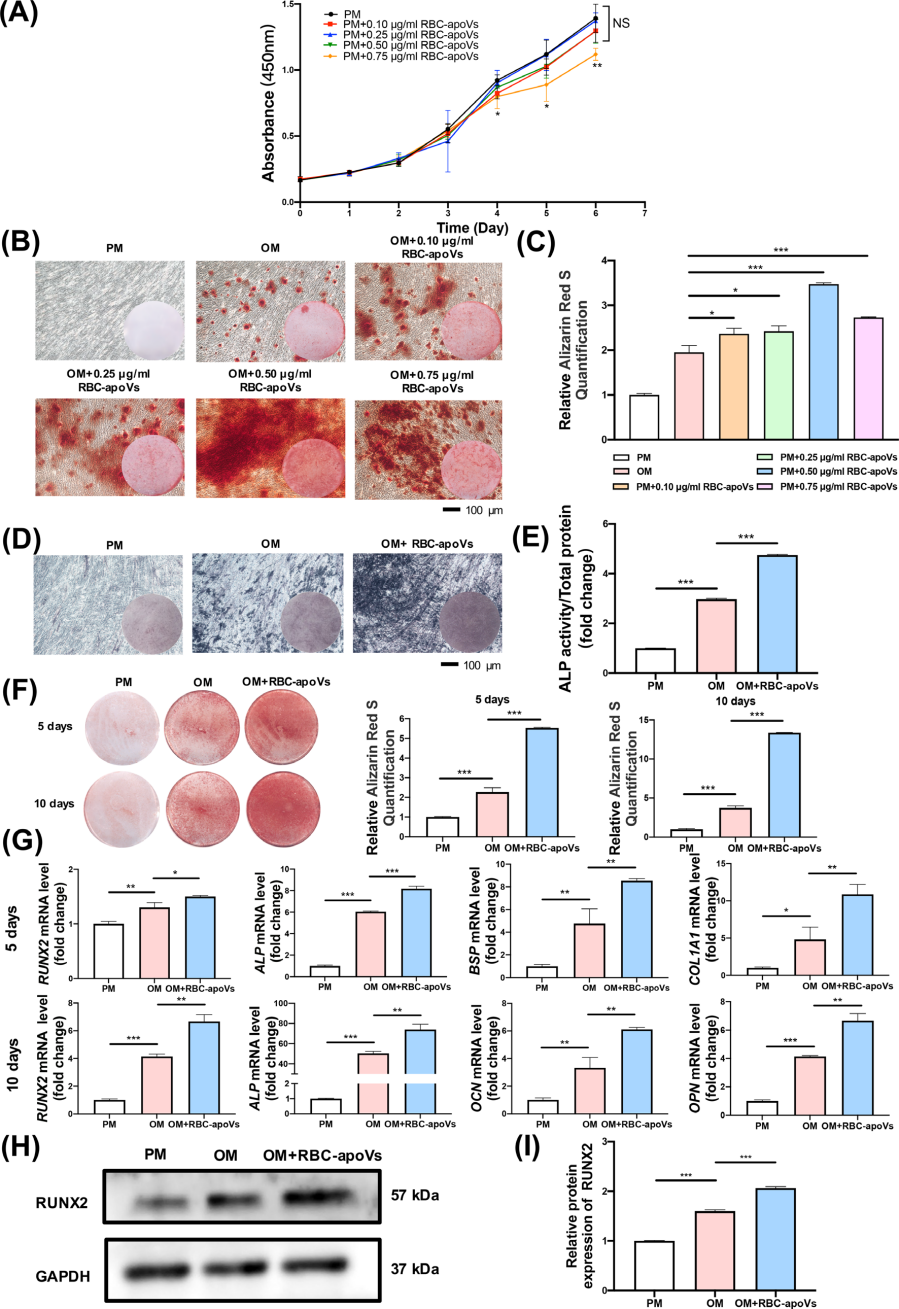
RBC‐apoVs promoted osteogenic differentiation of hBMSCs in vitro. (A) Growth curve measured by CCK‐8 assay; 0.75 μg/mL RBC‐apoVs inhibited cell proliferation of hBMSCs from Days 4 to 6. (B) RBC‐apoVs at 0.10, 0.25, 0.50 and 0.75 μg/mL accelerated mineralization in hBMSCs as indicated by ARS staining. RBC‐apoVs at 0.50 μg/mL had the most mineralized nodule formation. (C) RBC‐apoVs promoted osteogenesis, as indicated by ARS quantification. (D) RBC‐apoVs promoted osteogenesis, as indicated by ALP staining. (E) RBC‐apoVs promoted osteogenesis on Day 5, as indicated by ALP activity quantification. ALP activity was normalized against the total protein content. (F) Effects of RBC‐apoVs on the osteogenic differentiation of hBMSCs at different time points, as indicated by ARS staining and quantification. (G) RBC‐apoVs promoted the expression of *RUNX2*, *ALP*, *BSP* and *COL1A1* on Day 5 and that of *RUNX2*, *ALP*, *OCN* and *OPN* on Day 10 of the incubation, as indicated by qRT‐PCR. (H) Western blot analysis of RUNX2, GAPDH as an internal control. (I) Western blot quantification showing the protein expression level of RUNX2 in Figure 4H. All data are presented as mean ± SD, *n* = 3. *p < 0.05, **p < 0.01 and ***p < 0.001. ALP, alkaline phosphatase; ARS, alizarin red S; hBMSCs, human bone marrow mesenchymal stem cells; OM, osteogenic media; PM, proliferation media; RBC‐apoVs, RBC‐derived apoVs.

We investigated how RBC‐apoVs affected the proliferation of hBMSC using the CCK‐8 assay. Adding RBC‐apoVs at concentrations of 0.10, 0.25 and 0.50 μg/mL had no significant influence on the proliferation ability of hBMSCs. However, when adding RBC‐apoVs at the concentration of 0.75 μg/mL, the cell proliferation capacity of hBMSCs was partially suppressed by RBC‐apoVs from Day 4 to 6 compared with hBMSCs cultured in PM only (Figure 4A). To determine the optimal concentration of RBC‐apoVs for osteoinductive activity, we cultured the hBMSCs in PM, OM and OM containing various concentrations of RBC‐apoVs (0.10, 0.25, 0.50 and 0.75 μg/mL) for 10 days. ARS staining and ARS quantification results revealed that RBC‐apoVs increased mineralized nodules formation compared with the OM group, peaking at 0.50 μg/mL (Figure 4B,C). Therefore, 0.50 μg/mL RBC‐apoVs was chosen in the subsequent experiments. To validate the influence of RBC‐apoVs on osteogenic differentiation, we cultured the hBMSCs in OM with or without RBC‐apoVs for 5 and 10 days. Both ALP staining and ALP activity quantification results indicated that RBC‐apoVs could increase ALP activity at 5 days (Figure 4D,E). RBC‐apoVs were able to enhance the osteogenic differentiation of hBMSCs in OM after induction for 5 and 10 days (Figure 4F). Moreover, in hBMSCs treated with OM and RBC‐apoVs, the mRNA expression level of the osteogenic markers *ALP*, *RUNX2*, *COL1A1* and *BSP* increased significantly after 5 days and that of *ALP*, *RUNX2*, *OCN* and *OPN* after 10 days (Figure 4G). Western blotting results revealed that RUNX2 protein expression was elevated by RBC‐apoVs (Figure 4H,I). Therefore, RBC‐apoVs could promote the osteogenesis of hBMSCs in vitro.

### 3.4 RBC‐apoVs promoted osteogenesis of hBMSCs in vivo

**FIGURE 5:**
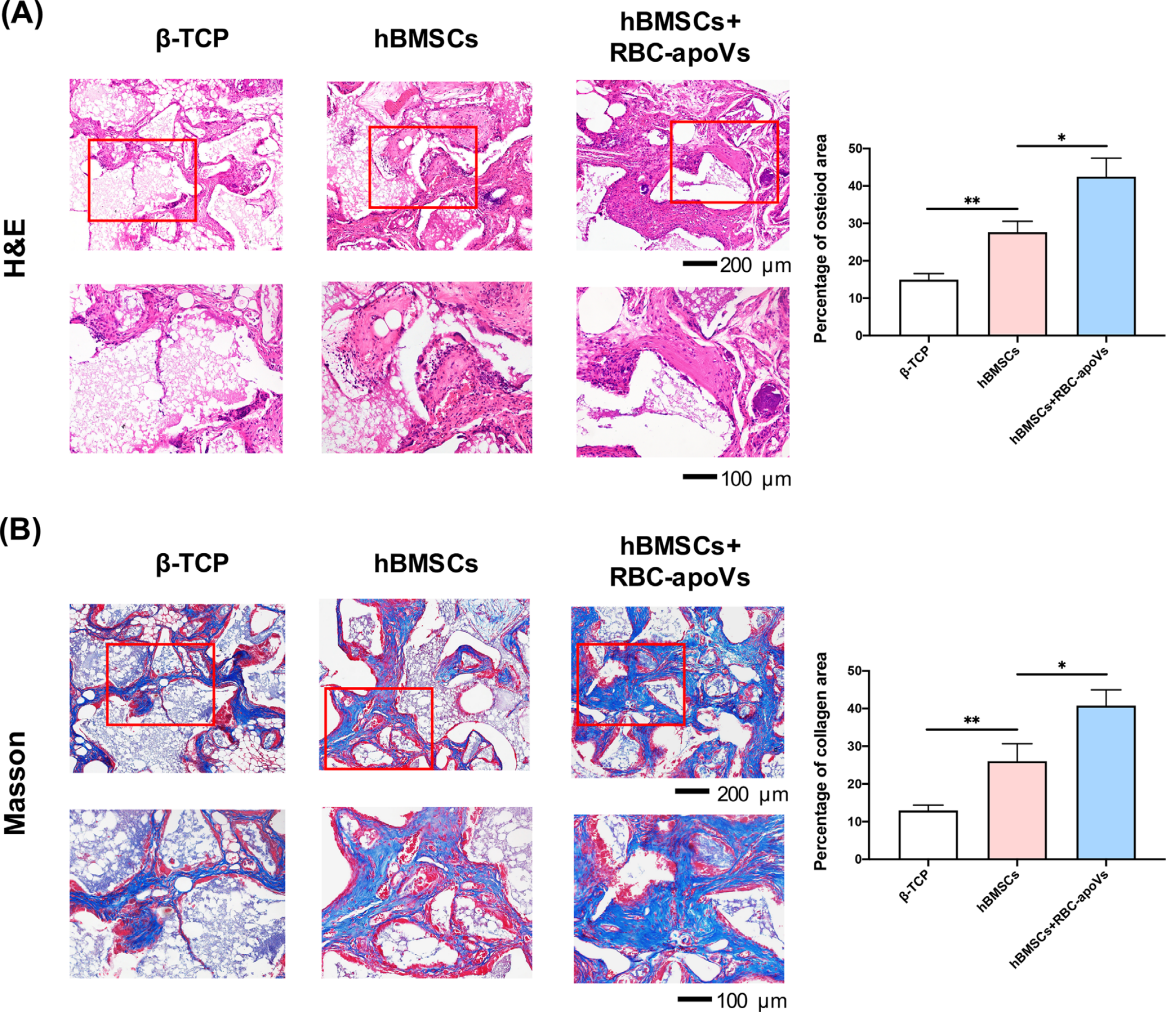
RBC‐apoVs promoted osteogenic differentiation of hBMSCs in vivo. (A) H&E staining and the corresponding quantification of the neo‐generated tissues. (B) Masson staining and the corresponding quantification of the collagen area. The red rectangles indicated the corresponding magnified areas. The data are presented as the mean ± SD, *n* = 3. *p < 0.05, **p < 0.01. ﻿﻿β‐TCP, β‐tricalcium phosphate; hBMSCs, human bone marrow‐derived mesenchymal stem cells; H&E, haematoxylin–eosin; RBC‐apoVs, RBC‐derived apoVs.

Given our findings in vitro, we investigated the impact of RBC‐apoVs on the osteogenesis of hBMSCs in vivo. hBMSCs were cultured in PM (hBMSCs group) and PM with RBC‐apoVs (hBMSCs + RBC‐apoVs group) for 7 days. We loaded the cells onto β‐TCP to implant them into nude mice. More newly‐formed eosinophilic bone‐like tissues were found in the hBMSCs + RBC‐apoVs group than in the β‐TCP group and the hBMSCs group, as shown by H&E staining results (Figure 5A). Masson staining results showed more collagen fibres (blue stained) in the hBMSCs + RBC‐apoVs group compared with the other groups (Figure 5B). Collectively, RBC‐apoVs promoted osteogenesis of hBMSCs in vivo.

### 3.5 RBC‐apoVs rescued critical‐sized rat calvarial defects in vivo

**FIGURE 6:**
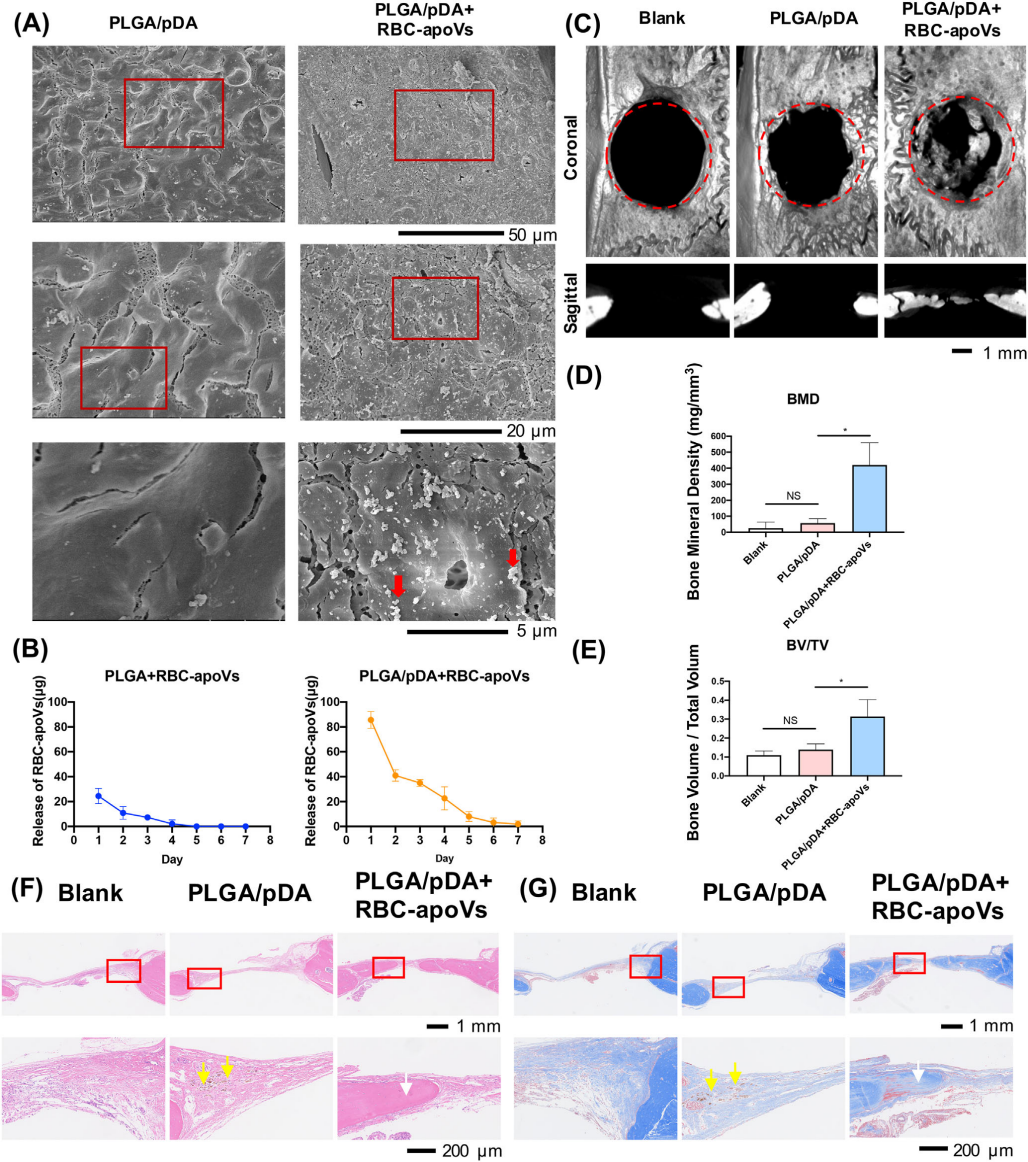
Therapeutic effect of RBC‐apoVs in a critical‐sized rat calvarial defect model. (A) SEM of PLGA/pDA scaffolds (PLGA/pDA) and PLGA/pDA with RBC‐apoVs (PLGA/pDA + RBC‐apoVs). The red rectangles indicated the corresponding magnified areas. The red arrows represented the RBC‐apoVs. (B) In vitro RBC‐apoVs release in stroke‐physiological saline from RBC‐apoVs‐loaded PLGA (PLGA + RBC‐apoVs) and RBC‐apoVs‐loaded PLGA/pDA (PLGA/pDA + RBC‐apoVs). (C) Micro‐CT images of bone formation. (D) Quantitative comparison of the bone mineral density (BMD) and (E) the bone volume/total volume (BV/TV) among different groups. The data are shown as the mean ± SD, *n* = 3. *p < 0.05. (F) H&E staining and (G) Masson staining of the Blank, PLGA/pDA, and PLGA/pDA + RBC‐apoVs groups. The red rectangles represented the corresponding magnified areas. The brown inclusions marked by the yellow arrows were the remaining PLGA/pDA. The white arrows indicated the newly formed bone tissue. H&E, haematoxylin‐eosin; PLGA, poly(lactic‐co‐glycolic acid); PLGA/pDA, PLGA scaffolds coated with polydopamine; RBC‐apoVs, RBC‐derived apoVs; SEM, scanning electron microscopy.

To further investigate the therapeutic impact of RBC‐apoVs, the local application of RBC‐apoVs was tested. In our previous study, PLGA scaffolds coated with polydopamine (PLGA/pDA) were successfully applied to combine and slowly release exosomes in mouse calvarial defects.^6^ In this study, we combined PLGA/pDA scaffolds and RBC‐apoVs to construct a cell‐free system. PLGA and PLGA/pDA were prepared as previously described and combined with RBC‐apoVs. The morphology of PLGA/pDA and PLGA/pDA with RBC‐apoVs was analysed by SEM, showing successful adhesion of RBC‐apoVs on the surface of PLGA/pDA scaffolds (Figure 6A). To verify the adhesive and slow‐release properties of PLGA/pDA, we tested the in vitro release of RBC‐apoVs in stroke‐physiological saline from RBC‐apoVs‐loaded PLGA and PLGA/pDA, respectively. The results indicated that PLGA/pDA not only absorbed more RBC‐apoVs than the PLGA scaffold but also released RBC‐apoVs in a slower pattern (Figure 6B). Therefore, PLGA/pDA scaffolds were appropriate for the local application of RBC‐apoVs.

A critical‐sized rat calvarial defects model was established. We set a blank group and implanted either PLGA/pDA or PLGA/pDA with RBC‐apoVs (PLGA/pDA + RBC‐apoVs) in the other two groups. Micro‐CT images revealed that the defects in the blank group and the PLGA/pDA group almost had no healing, while high‐density new bone could be found along the borders of the defect in the PLGA/pDA + RBC‐apoVs group (Figure 6C). Bone morphometric quantification indicated that RBC‐apoVs caused considerable bone formation, as shown by the increase of the bone mineral density (BMD) and the bone volume/total volume (BV/TV) of the PLGA/pDA + RBC‐apoVs group (Figure 6D,E). H&E staining showed only fibrotic tissue in the defect areas of the blank group. In the PLGA/pDA group, both fibres and the remaining PLGA/pDA material could be identified. More new bone tissue was detected in the PLGA/pDA + RBC‐apoVs group than in the other groups (Figure 6F). More blue‐stained collagen formations could be found in the PLGA/pDA + RBC‐apoVs group than those in the other two groups, as shown by the Masson staining (Figure 6G).

### 3.6 Proteomic profiling of hBMSCs treated with RBC‐apoVs

**FIGURE 7:**
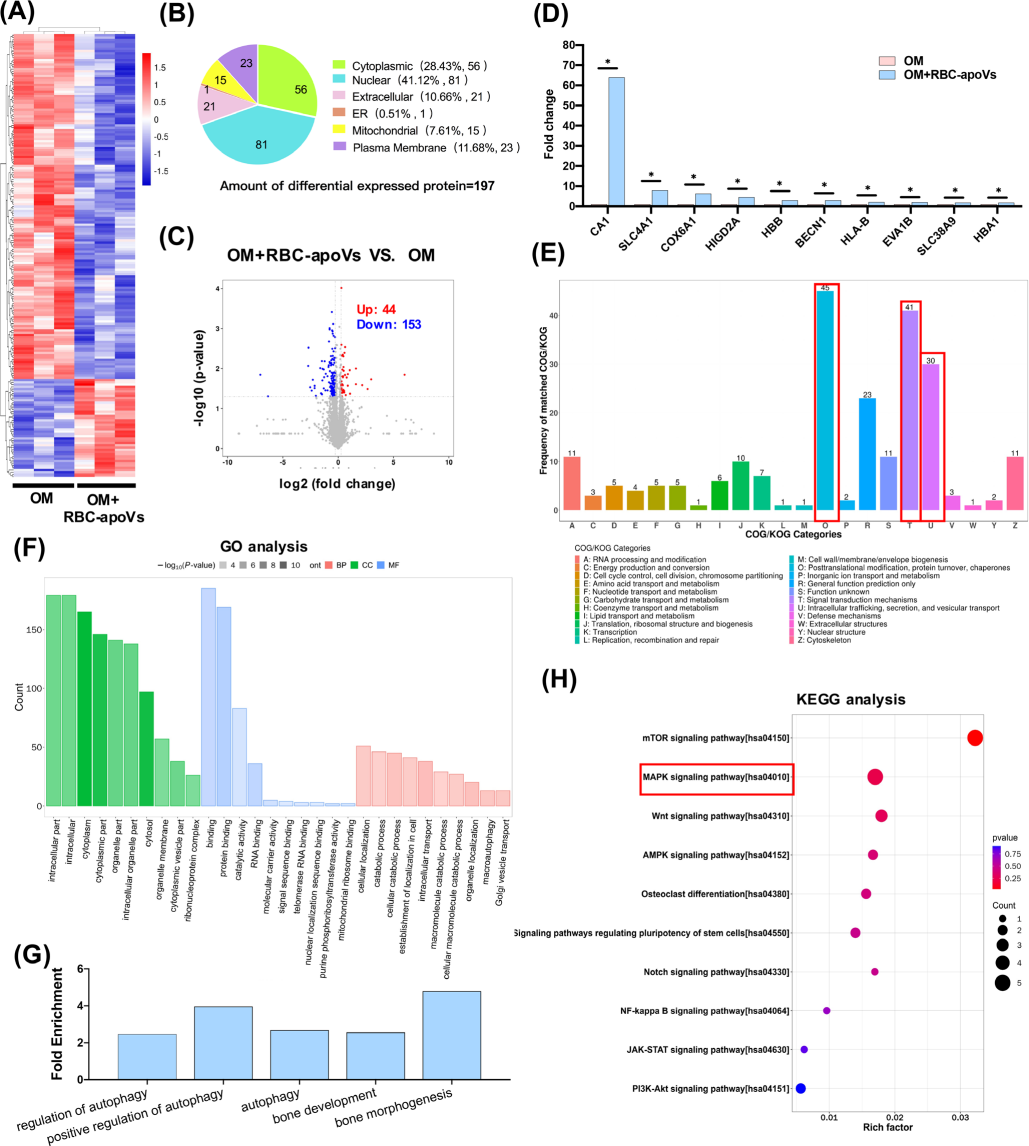
Proteomic analysis of hBMSCs treated with OM and OM + RBC‐apoVs. (A) Hierarchical clustering of the differentially expressed proteins (DEPs; fold change < 0.83 or >1.2; *p* value < 0.05) between the OM group and the OM + RBC‐apoVs group. Rows represent proteins and columns represent individual replicates. Enrichment is depicted in red and depletion in blue. (B) Subcellular localization of the DEPs. ER, endoplasmic reticulum. (C) Volcano plots showing significantly upregulated (red dots) and downregulated (blue dots) proteins in the OM + RBC‐apoVs group compared with the OM group. (D) The top 10 upregulated proteins with the greatest protein expression fold change in the OM + RBC‐apoVs group compared with the OM group. CA1 had the largest fold change among the upregulated proteins. *p < 0.05. (E) Clusters of orthologous groups for eukaryotic complete genomes (KOG) analysis of the DEPs. Most of the DEPs belonged to the ‘Posttranslational modification, protein turnover, chaperones’, ‘Signal transduction mechanism’ and ‘Intracellular trafficking, secretion and vesicular transport’ categories, as marked by the red rectangles. (F) Gene ontology (GO) analysis of DEPs in the OM + RBC‐apoVs group compared with the OM group, categorized into the ‘Cellular component (CC)’, ‘Molecular function (MF)’ and ‘Biological process (BP)’ domains. (G) Fold enrichment of GO terms related to the autophagy and osteogenesis of hBMSCs treated with RBC‐apoVs. (H) Kyoto Encyclopedia of Genes and Genomes (KEGG) pathway analysis of pathways related to the osteogenesis of hBMSCs treated with RBC‐apoVs. MAPK pathway showed a high enrichment, as indicated by the red rectangle. DEPs, differentially expressed proteins; hBMSCs, human bone mesenchymal stem cells; OM, osteogenic induction medium; RBC‐apoVs, RBC‐derived apoVs.

To identify the specific proteomic changes of RBC‐apoVs brought to hBMSCs during the osteogenesis process, we cultured hBMSCs in (OM, OM + RBC‐apoVs) for 10 days, prepared their protein and performed LC–MS/MS analysis. The result showed that among the 5151 proteins that were identified, there were 197 differentially expressed proteins (DEPs) between the OM group and the OM + RBC‐apoVs group (Figure 7A). The subcellular localizations of DEPs were mainly in the nucleus, cytoplasm, plasma membrane and extracellular space (Figure 7B). In the OM+ RBC‐apoVs group, 44 proteins had significantly higher levels compared to the OM group, whereas 153 proteins were downregulated (Figure 7C). The top 10 upregulated proteins with the largest fold change were presented (Figure 7D). Among them, CA1 had the highest expression level change, which increased by 63.971 times in hBMSCs of the OM + RBC‐apoVs compared with those in the OM group. CA1 is a type of zinc metalloenzyme mainly involved in catalysing the reversible hydration reaction of carbon dioxide, which is abundant in RBCs.^32^ Notably, the expression levels of several proteins closely related to RBCs increased significantly in the OM + RBC‐apoVs group, including haemoglobin subunit alpha 1 (HBA1), solute carrier family 4 member 1 (SLC4A1), and haemoglobin subunit beta (HBB). The above results indicated that RBC‐related protein components were probably transported into hBMSCs by RBC‐apoVs, which led to their enrichment in target cells. In addition to the upregulated expression of protein related to RBCs, the expression of some other functional proteins was also significantly increased, such as the autophagy‐related protein Beclin 1 (BECN1).

To further explore the function of the DEPs, we carried out the functional analyses. The KOG analysis was the homologous classification of gene products to identify the source and functional classification of DEPs. The results indicated that the DEPs could be classified into 22 categories, and they were mainly in the ‘Posttranslational modification, protein turnover, chaperones’, ‘Signal transduction mechanism’ and ‘Intracellular trafficking, secretion and vesicular transport’ categories (Figure 7E). The KOG results suggested extensive changes in the life activities of hBMSCs after adding RBC‐apoVs. As for GO annotations analysis, the DEPs were categorized into various terms in three main domains, including ‘cellular component’, ‘molecular function’ and ‘biological process’ (Figure 7F). Moreover, the DEPs were associated with autophagy‐related terms, as well as the ‘bone development’ term and the ‘bone morphogenesis’ term, which were related to the bone formation process (Figure 7G, Table S1). KEGG pathway analysis suggested that the DEPs were enriched in multiple KEGG pathways. Specifically, the signalling pathways related to osteogenesis were identified (Figure 7H). These findings paved the way for subsequent research into the molecular mechanism underlying the effect of RBC‐apoVs on the osteogenic differentiation of hBMSCs.

### 3.7 RBC‐apoVs promoted osteogenesis of hBMSCs via CA1 and P38 pathway

**FIGURE 8:**
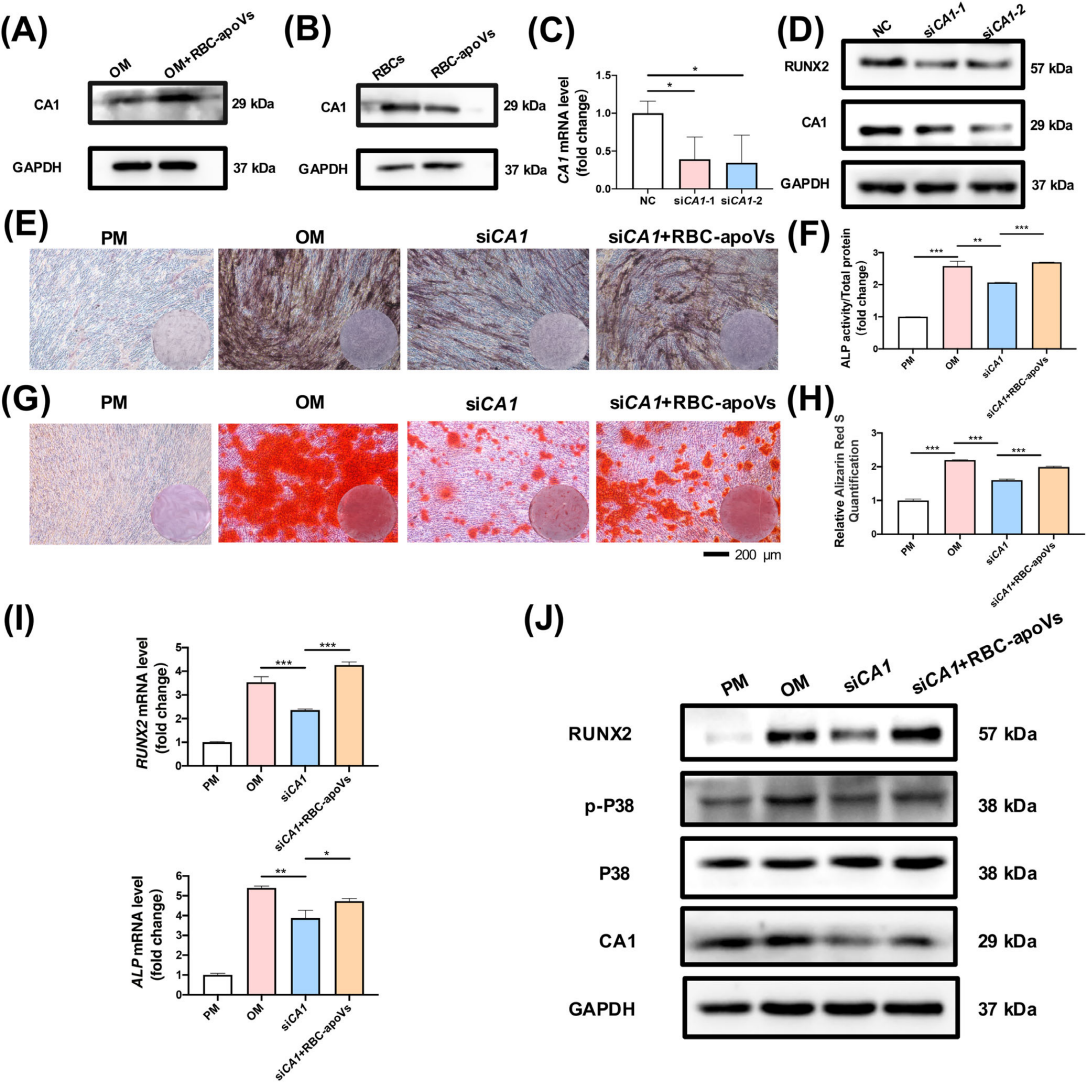
RBC‐apoVs promoted osteogenesis of hBMSCs via CA1. (A) Western blot analysis of CA1 and the internal control GAPDH. The hBMSCs were cultured in OM and OM with RBC‐apoVs for 10 days. (B) Western blot analysis of CA1 and the internal control GAPDH. (C) *CA1* mRNA expression level detected by qRT‐PCR. (D) CA1 protein expression level detected by Western blot. (E) Knockdown of *CA1* inhibited osteogenesis, while RBC‐apoVs ameliorated osteogenesis of hBMSCs, as indicated by ALP staining. The hBMSCs were cultured in PM and OM, and *CA1* knockdown hBMSCs were incubated with OM (si*CA1*) and OM with RBC‐apoVs (si*CA1* + RBC‐apoVs). (F) ALP activity quantification results, consistent with the result of ALP staining. (G) Knockdown of *CA1* inhibited osteogenesis, while RBC‐apoVs ameliorated osteogenesis of hBMSCs, as indicated by ARS staining. (H) ARS quantification results, consistent with the result of ARS staining. (I) Suppressing the expression of *CA1* inhibited the mRNA expression of *ALP* and *RUNX2*, as indicated by RT‐qPCR. (J) Western blot analysis of protein expression of RUNX2, p‐P38, P38 and CA1. GAPDH was the internal control. All data are presented as mean ± SD, *n* = 3. *p < 0.05, **p < 0.01 and ***p < 0.001. GAPDH, glyceraldehyde‐3‐phosphate dehydrogenase; hBMSCs, human bone mesenchymal stem cells; OM, osteogenic induction medium; RBC‐apoVs, RBC‐derived apoVs.

According to the proteomic analysis results, RBC‐apoVs may have the ability to transfer RBC‐related proteins into hBMSCs and influence the osteogenesis progress. Among the upregulated proteins, CA1 had the most significant fold change of protein expression in the OM + RBC‐apoVs group compared with the OM group. CA1 has been suggested to correlate with the bone formation of Saos‐2 cells (human osteogenic sarcoma cells).^35^ However, the relationship between CA1 and the osteogenesis of hBMSCs was unclear. Therefore, we focused on the enriched protein CA1 in specific and further explored its role in the osteogenesis of hBMSCs. We performed Western blot analysis to elucidate whether adding RBC‐apoVs could influence the protein level of CA1 in hBMSCs. Consistent with the findings of the proteomic analysis, the protein level of CA1 increased in the OM + RBC‐apoVs group compared to the OM group (Figure 8A). Western blot results also proved the high expression level of CA1 in both RBCs and RBC‐apoVs (Figure 8B). Next, we used small interfering RNA (siRNA) to suppress CA1 expression in hBMSCs. We performed qRT‐PCR and Western blot analysis to confirm the efficiency of CA1 silencing (Figure 8C,D). Then, we cultured the CA1 knockdown cells in the osteogenic medium with (the si*CA1* + RBC‐apoVs group) or without RBC‐apoVs (the si*CA1 group*). ALP staining and activity quantification demonstrated that the depletion of CA1 inhibited the osteogenic differentiation of hBMSCs, while this effect could be partially ameliorated by adding RBC‐apoVs into CA1 knockdown cells (Figure 8E,F). These findings were verified by ARS staining and quantification (Figure 8G,H). Furthermore, *RUNX2* and *ALP* expression levels in the si*CA1* group were significantly lower than those in the OM group, whereas the presence of RBC‐apoVs increased the mRNA expression levels (Figure 8I). Western blot analysis showed a decrease in the protein expression level of CA1 and RUNX2 during osteogenesis in CA1 knockdown hBMSCs, whereas RBC‐apoVs promoted the expression. Moreover, the P38 MAPK signalling pathway was suppressed in the si*CA1* group compared with the OM group, while the P38 phosphorylation increased in the si*CA1* + RBC‐apoVs group. These results suggested that RBC‐apoVs possibly regulated osteogenesis through the P38 MAPK pathway, which corresponded with the proteomic analysis results (Figures 7H and 8J). Collectively, the above results demonstrated that CA1 was a positive regulator of osteogenic differentiation, and RBC‐apoVs could regulate the osteogenesis of hBMSCs via CA1 and the P38 MAPK pathway.

## 4 DISCUSSION

RBCs are the most numerous type of cell in the body. Previous studies indicated that RBCs could produce vesicles in physiological and pathological conditions, showing their potential as a promising cell source for EV production. In recent years, researchers have paid attention to the biological characteristics and functions of RBC‐derived EVs. Since mature RBCs do not contain nuclei and organelles, they do not transfer genetic materials like DNA to the recipient cells during the production and function processes of RBC‐derived EVs, resulting in a low risk of neoplastic transformation and a high level of biological safety. In addition, proteomic and transcriptomic studies have shown that RBC‐derived EVs contain a variety of proteins and miRNAs despite the lack of DNA components.^21^, ^26^, ^36^, ^37^ Because of their short life span, RBCs frequently undergo apoptosis in physiological homeostasis maintenance of the human body. RBCs have similar biological behaviours during apoptosis, such as cell shrinkage, membrane blebbing and protrusion, and the exposure of PS.^25^, ^38^ In previous studies, EVs were often extracted from RBCs stored in vitro. Several drugs, such as calcium ionophores, were used to treat RBCs for more than 12 h in order to stimulate RBCs' death and produce vesicles.^31^, ^39^, ^40^, ^41^ Here, we separated apoVs from RBCs that were freshly obtained from human venous blood after apoptosis induction. We revealed the size and morphology of the RBC‐apoVs and showed that they were abundant in EV‐specific markers, CD81 and TSG101.^42^ The high positive rate of Annexin V obtained by flow cytometry showed that these vesicles had exposure of PS, which is a characteristic of apoVs.^43^ We also found that RBC‐apoVs inherited the surface signature of their parental cells, such as CD235a. These findings suggested an efficient way of producing RBC‐apoVs.

ApoVs are an attractive alternative treatment for multiple diseases. They possess many advantages over exosome therapy, especially their higher yield, simpler extraction methods, and lower costs.^15^, ^44^ The application of apoVs in the field of tissue repair has drawn more and more attention. Bone tissue engineering can repair bone defects resulting from trauma, congenital malformation, inflammation and tumours, which are commonly seen in clinics. The osteogenic differentiation of stem cells is crucial for bone tissue engineering.^45^ Studies of apoVs in bone regeneration provide a low‐risk and low‐cost approach to cell‐free bone tissue engineering.^19^ BMSCs have been found to undergo apoptosis within 2 days after transplantation into a bone defect, and the apoVs isolated from BMSCs can promote the healing of the defect.^46^ In addition, it has been reported that mature osteoclast‐derived apoptotic bodies could promote the osteogenic differentiation of MC3T3‐E1 cells, showing transcriptome and functional similarities to their parental cells.^47^, ^48^ These studies suggested that apoVs have a potential role in bone tissue repair. However, the parental cells of the above apoVs could not be extracted from patients in large quantities, so the cells had to be extracted or purchased in advance, which could raise ethical concerns. Cell expansion in vitro was required before apoptotic induction.^19^ Besides, MSC‐apoVs still have limitations in biological safety because they contain DNA that could be transferred to recipient cells. Therefore, we intended to seek a more efficient and safer alternative source of apoVs. RBCs can be immediately obtained from patients with little harm, so cell expansion is not necessary. Due to their unique cellular structures, RBCs can be induced to undergo apoptosis by using RBC lysis buffer for 30 min instead of using cytotoxic drugs such as STS overnight, which is harmless to nucleated cells and time‐saving. In addition, RBCs do not transfer genetic material into recipient cells, showing a higher level of biological safety. Collectively, our study has provided a method for producing RBC‐apoVs that could promote the clinical translation of apoVs.

In the process of bone defect, the vascular injury in the defect area is accompanied by the damage and apoptosis of RBCs. During the repair of bone defects, angiogenesis occurs and numerous RBCs travel through the defect areas via the blood flow.^49^ However, previous research did not explore the influence of apoVs derived from RBCs on bone formation and stem cell osteogenesis. Our study indicated that RBC‐apoVs could be ingested by hBMSCs. We investigated that RBC‐apoVs could promote the osteogenesis of hBMSCs in vitro and in vivo. Besides, RBC‐apoVs exerted therapeutic effect on the rat calvarial defect. Nevertheless, the promoting effect of RBC‐apoVs was weakened when the concentration exceeded a certain level. Combining with the CCK8 results that the proliferation of hBMSCs was inhibited by RBC‐apoVs at high concentrations, our findings suggested that the concentration of RBC‐apoVs needed to be controlled in therapeutic application. Moreover, the capability of RBC‐apoVs to enhance osteogenesis at different stages of the osteogenesis process has not been explored in depth, so further research is needed.

The mechanism by which RBC‐apoVs affect osteogenesis was elucidated. Previous studies have confirmed that the components of apoVs depend on their parental cells, and some proteins could be inherited from parental cells by apoVs.^15^, ^44^ In this study, the proteomic analysis results showed significant enrichment of proteins related to RBCs in the OM + RBC‐apoVs group compared with the OM group, such as CA1, SLC4A1, and HBA. The existence of these proteins indicated that RBC‐apoVs inherited some of the components from RBCs and transported the protein components into hBMSCs successfully. Among the differentially expressed proteins, the one with the largest fold change in expression difference is the CA1 protein, which is found at the highest level in RBCs but at a low level in stem cells. CA1 can catalyse the reversible hydration of carbon dioxide, producing bicarbonate and hydronium ion. Several studies show that it participates in a variety of biological processes.^50^, ^51^ It has been reported that CA1 may be related to calcification and bone formation.^35^, ^52^ Inhibitors of carbonic anhydrase also hinder the bone formation of Saos‐2 cells, while the carbonic anhydrase activators can promote biomineralization.^35^, ^53^ However, previous studies have only explored that CA1 can promote calcification and bone formation in tumour cell lines, and there has been no information about the effect of CA1 on stem cells. Western blot analysis showed that RBC‐apoVs transported CA1 into hBMSCs, confirming the proteomic findings. We further confirmed the regulatory effects of RBC‐apoVs on osteogenic differentiation of hBMSCs by depletion of CA1. Knockdown of CA1 inhibited the osteogenesis of hBMSCs, as indicated by ALP and ARS staining. RBC‐apoVs partially ameliorated the weakened osteogenic differentiation of hBMSCs caused by CA1 knockdown. The findings were strengthened by mRNA and protein expression analyses of bone formation factors. Taken together, our results demonstrated that RBC‐apoVs could promote the osteogenesis of hBMSCs, and CA1 played a positive role in this process. The influence of CA1 on bone formation in vivo requires additional study. Meanwhile, we found that the P38 MAPK signalling pathway was upregulated when RBC‐apoVs were added, which was consistent with the KEGG analysis results. However, more in‐depth research on the underlying mechanism is still needed. Other bioactive cargoes, such as miRNA, may play a role in the therapeutic effect of RBC‐apoVs. The proteomic analysis results also suggested that RBC‐apoVs might affect the osteogenic differentiation of hBMSCs in more than one way. BECN1 (Beclin 1), a crucial protein in autophagy,^54^ was significantly upregulated in hBMSCs of the OM + RBC‐apoVs group in comparison to the cells in the OM group (Figure 7D). Previous research has demonstrated that autophagy positively influences the osteogenesis of human MSCs.^55^ Therefore, it is possible that RBC‐apoVs may have beneficial effect on hBMSCs through the regulation of autophagy. Further evaluation of the relation between RBC‐apoVs' influence on autophagy and the osteogenesis of hBMSCs needs to be done.

Overall, our results showed that RBC‐apoVs could be extracted efficiently. RBC‐apoVs could promote the osteogenesis of hBMSCs and enhance bone defect healing, suggesting their potential for therapeutic application in bone tissue engineering. However, our study still has limitations. It is known that mature RBCs have no nuclei. Although our current results suggest that RBC‐apoVs were able to deliver CA1 into the recipient cells, which in turn played a role in promoting osteogenesis, the proof of the effect of the CA1 from RBC‐apoVs in osteogenesis was still not sufficient due to the technical constraints that we could not knock down CA1 in RBCs by common transfection methods.^31^, ^56^ The mechanism can be further studied with CA1‐depleted RBC‐apoVs, which requires more techniques in modifying the cargoes of RBC‐apoVs. Besides, further research in large animals is required to promote clinical translation.

## 5 CONCLUSION

ApoVs derived from RBCs could promote the osteogenesis of hBMSCs. RBC‐apoVs promoted bone regeneration in the calvarial bone defects. Mechanistically, RBC‐apoVs could transport CA1 and regulate the osteogenic differentiation of hBMSCs. Our findings indicated that RBC‐apoVs could provide a novel strategy in bone tissue engineering.

## AUTHOR CONTRIBUTIONS

Xiao Zhang, Yongsheng Zhou and Yuzi Shao initially designed the study. Yuzi Shao, Yuhe Jiang, Kunkun Yang and Yuan Zhu performed the experiments and collected the data. Yuzi Shao and Xiao Zhang did the job of data analyses and interpretation. Yunsong Liu, Ping Zhang, Longwei Lv, Xiao Zhang and Yongsheng Zhou contributed to the study conception and design and financial support. Yuzi Shao, Xiao Zhang and Yongsheng Zhou were responsible for manuscript writing and revision. All authors read the final manuscript and consented to the submission.

## FUNDING INFORMATION

This study was supported by grants from the Beijing Natural Science Foundation (No. 7222224) and the National Natural Science Foundation of China (No. 81930026).

## CONFLICT OF INTEREST STATEMENT

The authors declare no conflicts of interest.

## Supporting information


**Table S1.** Proteins associated with bone development and bone morphogenesis.

## Data Availability

The data generated or analyzed in this study are available from the corresponding authors on reasonable request.
